# Supplementary material for: Are carbon emissions trading and green financial instruments synergistic? -Comprehensive quantitative research based on content analysis
Source: PLoS One. 2024 Mar 7;19(3):e0298601. doi: 10.1371/journal.pone.0298601 (PMC10919601; doi:10.1371/journal.pone.0298601)
Supplement: S1 Data — (ZIP) [file pone.0298601.s001.zip › Data/地级市Z_F工作报告.zip!/╔┬╬≈/▒a╝a╩╨/└┤╫╘╣1⁄2╓┌║┼í╛┬φ┐╦╩2╛▌═°í┐_2018.docx]

各位代表：

现在，我代表市人民政府向大会作工作报告，请予审议，并请市政协委员和其他列席人员提出意见。

2017年工作回顾

过去的一年，面对复杂严峻的经济形势，市政府聚焦追赶超越，坚持强优势与补短板并重，稳定传统动能与创新发展并举，真抓实干，主动作为，较好完成了市十五届人大一次会议确定的主要目标任务。全市实现地区生产总值2179.81亿元，增长8.7%，三次产业结构比达到8∶64.5∶27.5；完成财政总收入209.49亿元，增长18%，地方财政收入达到81.08亿元，同口径增长9.7%；城乡居民人均可支配收入达到34351元和11209元，分别增长8.3%和9%，全面建设最具幸福感城市和关天副中心城市迈出坚实步伐。

强化产业支撑，经济实力不断增强。工业强市“1553”行动计划加快推进，吉利36万台汽车发动机下线，汽车整车产量达到19.4万辆，法士特100万台中轻型变速器、陕汽控股全系列商用车暨新能源基地等项目顺利开工，90万吨甲醇、7107航天基地、蔡家坡百万平方米标准化厂房等一批重大项目加快建设，麟游低热值发电厂即将建成，华润丰台山100兆瓦风电项目并网发电，宝鸡钛谷钛产业战略研究院及军民融合产业联盟成立，机器人智能制造产业园成为全省示范。规模以上工业总产值和全市工业增加值分别突破三千亿元和一千亿元大关。特色农业发展走在全省前列，陕果集团万亩猕猴桃和苹果示范基地启动建设，陇县羊乳产业加快发展，6个优势农产品加工园区快速推进，成功举办世界猕猴桃大会、全国蜂业大会，千阳被评为中国苹果强县。全市新发展苗木花卉7.8万亩，苹果、猕猴桃面积分别达到72.5万亩和36.8万亩。旅游产业持续走高，全年接待游客7986.5万人次，实现旅游综合收入579.6亿元，分别增长25.1%和31.8%。九龙山景区建成开园，金台观创建为国家4A级景区，太白山、关山草原、渭河百里画廊等景点景观全面提升，《法门往事》成功投演，太白鳌山滑雪场等3个景区入选《全国优选旅游项目名录》，凤县革命纪念馆入列全国红色旅游经典景区。消费市场保持增势，社会消费品零售总额达到800.9亿元，增长14.1%。东岭国金中心竣工投用，银泰购物中心、洲际皇冠假日酒店建成开业，胜利塬封神演义文化主题公园等一批夜间经济示范点投入运营。大数据等新经济、新业态快速发展，渭滨互联网产业园获评全国青年创业示范园区,我市荣膺“全国优秀电子商务城市”称号。

加强基础建设，城市品质明显改善。《幸福宝鸡规划》加快实施，联盟、钛谷等8座渭河大桥，以及虢磻路铁路桥、金陵河中山桥等工程全面建设，虢镇南环路、陈仓中路、川陕路等综合管廊工程主体完工，10条城市道路改造提升和海绵城市建设进展顺利。新发展天然气用户2.36万户，新增集中供热面积348万平方米。完成城市“五拆”32.4万平方米，改造老旧小区163个，建成保障性住房2.59万套，棚户区改造和保障房建设实现全省“四连冠”。顺利通过全国文明城市、国家卫生城市、中国人居环境奖和国家节水型城市复检。重点区域开发提速，高新科技新城太公湖生态文化区开工建设，交大蟠龙医院建设加快推进，蔡家坡被命名为国家火炬重型汽车及零部件特色产业基地。实施县城改造提升项目325个，太白、凤县5A级景区县城建设积极推进，凤翔柳林镇、扶风法门镇跻身全国特色小镇。交通枢纽建设实现新突破，宝兰客专开通运营，坪坎至汉中高速建成通车，绛汤高速主体建成，陇县至陕甘界高速基本贯通，连霍高速过境线、太凤高速加快建设，凤翔至旬邑高速、西安至法门寺、法门寺至咸阳机场城际铁路顺利开工。

深化改革创新，市场活力进一步释放。“放管服”改革向纵深推进，项目在线申报、办结数跃居全省第一。“双随机、一公开”监管和“五证合一、一照一码”登记模式全面推行，市级行政许可事项总数减少61%，新增各类市场主体3.54万户。农村“三权分置”改革全面推进，积极实施“三变”改革试点。国企改革政策体系不断完善，宝纺集团搬迁技改、医药集团整合重组全面启动，“三供一业”分离移交步伐加快。创新能力持续增强，国家创新型城市建设通过评估，激光加工国家工程研究西北区域中心、国家数控系统工程技术研究中心陕西分中心投入运行，西北有色金属研究院宝鸡分院、特种飞行研究院挂牌成立，宝鸡高新区进入全国高新区“第一阵营”，在156个国家高新区排名中上升至第53位。投融资创新平稳推进，新引进金融机构4家，巨成钛业、晨光电器成功挂牌“新三板”。实施PPP项目29个，吸引社会资本51.39亿元。通过设立政府投资基金、加强与金融机构合作等方式，为36个重点项目筹集资金234.6亿元。对外开放步伐加快，钛产品、机床等4个基地入围全省特色出口基地，宝石钢管哈萨克斯坦合资项目投产运营,俄罗斯冰淇淋加工项目落户宝鸡。

铁腕治污降霾，生态建设持续发力。保卫蓝天“1+18”专项行动全面推进，在全省率先开展农村“五改”和环保过冬“三告别”，完成“五改”75.88万户，取缔改造“散乱污”企业113户，市区空气优良天数达到247天，连续4年位居关中第一。水污染治理扎实开展，全面推行河长制，实施第二轮渭河治污三年计划，开展渭河4条重点支流清水行动，基本实现直排渭河污水全截污、全收集、全处理。建成渭河生态公园14个、湖泊水面23个，渭河堤顶道路全线开通。绿色宝鸡建设持续深化，完成绿化提升37.5万亩，植树4190万株，千渭之会、眉县龙源国家湿地公园建成命名。生态创建成效明显，实现国家园林县城全覆盖，荣获全省唯一的“中国美丽乡村建设示范地区”称号。

大力改善民生，群众得到更多实惠。全市民生支出达到251.1亿元，占到一般公共预算的83.3%。聚力开展创业就业专项行动，城镇新增就业5.4万人，农村劳动力转移就业95.2万人。社保服务体系不断完善，率先在西北地区建立补充工伤保险制度，城乡居民养老保险“五个不出村”经办服务模式在全省推广，成为全国首批异地就医直接结算城市。教育现代化有力推进，实施教育基础建设项目129个，公办高中全部达到省级标准化高中，宝鸡文理学院创建宝鸡大学顺利推进，发放各类教育资助资金1.19亿元，惠及学生15.27万人次。医改宝鸡模式持续深化，入选全国新农合支付方式改革试点市、首批国家公立医院综合改革示范市，被国务院表彰为公立医院综合改革真抓实干成效明显先进市。举办国家和省级大型体育赛事13项，第九届陕西省艺术节申办成功。投资50多亿元的“十件实事”全面完成，281所学校体育设施免费向群众开放，农村公路“最后一公里”建成5334公里，养老服务体系建设和残疾人工作受到省政府表彰,眉县被评为首批全国幸福社区建设示范县。安全生产形势平稳，防震减灾、信访维稳、供销工作荣获全国先进，成功创建为首批国家食品安全示范城市。

与此同时，审计、质监、统计、物价、气象、档案、地志、人民防空、民族宗教、国家安全、妇女儿童、侨务外事、红十字会等工作也取得了新的成绩。

各位代表，回顾过去一年的工作，困难比预料的多，挑战比预想的大，但成绩比预计的好。一年来，我们始终坚持把加快发展作为第一要务，聚焦“两市”建设，加速追赶超越，GDP增长高于全国1.8个百分点，经济增速关中五市第一；始终坚持把项目建设作为关键举措，大力开展工业招商季活动，千方百计扩大有效投资，固定资产投资增速和招商引资工作走在全省前列；始终坚持把重大基础设施建设作为重要支撑，抓住李克强总理来宝考察机遇，综合保税区、机场等项目推进取得实质性进展；始终坚持把脱贫攻坚作为最大的民生工程，全方位强化精准施策、全覆盖抓好结对帮扶、全过程保持攻坚态势，全市177个贫困村退出、6.67万人脱贫，“旅游+扶贫”模式成为全省样板；始终坚持把转变工作作风作为重要保障，各级干部干事创业的风气日益浓厚，政府的执行力和服务水平不断提升，政风行风测评群众满意度排名全省前列。

各位代表，宝鸡一年来经济社会发展取得的进步，是习近平新时代中国特色社会主义思想正确引航的结果，是市委坚强领导的结果，是市人大、市政协监督支持和社会各界共同努力的结果，饱含了各位代表、各位委员的智慧和力量，凝聚了全市人民的心血和汗水。在此，我代表市政府，向人大代表、政协委员、各民主党派、各人民团体、社会各界人士，向驻宝企事业单位、人民解放军、武警驻宝部队全体官兵、公安干警，表示崇高的敬意和诚挚的感谢!

在总结成绩的同时，我们也清醒地看到,人民日益增长的美好生活需要和不平衡、不充分的发展之间的矛盾仍然十分突出：优势支柱产业规模还不大，产业转型升级和新旧动能转换步伐较慢；支撑发展的大项目、好项目谋划储备还不够，开放型经济、现代服务业发展水平不高；脱贫攻坚任务重，县域发展不够平衡，大气污染问题依然比较突出，民生领域还有不少短板；一些公务人员还存在慢作为、不作为的问题，政务服务、营商环境需要进一步优化提升。对这些问题，我们将认真对待，勇于担当，切实加以解决，绝不辜负全市人民的期望。

2018年主要工作

2018年是全面贯彻落实党的十九大精神的开局之年，也是决胜全面建成小康社会、实施“十三五”规划承上启下的关键一年。当前，我市正处在转变发展方式、优化经济结构、转换增长动力的攻关期，既面临着宏观调控与资源环境的双重约束、稳增长与调结构的双重压力、扩总量与提质量的双重任务，也面临着国家振兴实体经济、加快发展先进制造业和《关中平原城市群发展规划》颁布实施等一系列重大机遇。立足新时代、新征程，我们必须瞄准新目标、实现新发展，充分发挥“一带一路”重要节点城市和国家级战略平台的叠加优势，抢抓经济转型升级的重要“机遇期”和“窗口期”，凝心聚力，乘势而上，奋力谱写新时代中国特色社会主义的宝鸡篇章。

按照市委十二届三次全会总体部署，今年政府工作的总体思路是：以党的十九大、中央经济工作会和省、市委全会精神为指导，全面落实“五新”战略，抢抓新时代历史机遇，聚焦追赶超越和高质量发展，坚持稳中求进、质量第一、效益优先、项目支撑和创新驱动，围绕打造副中心、建设最具幸福感城市总目标，着力发展枢纽经济、门户经济、流动经济，持续提升产业集群、物流枢纽、开放高地、人文凝聚、综合服务五大功能，突出精准脱贫、污染防治、风险防控、优化营商环境四项重点工作，加快质量、效率和动力三大变革，抓重点、补短板、强弱项，努力建设装备制造名城、历史文化名城、“一带一路”上国际化城市和宜居宜业美丽幸福城市。

经济社会发展的主要预期目标是：地区生产总值增长8.5%，全社会固定资产投资增长13%，规模以上工业增加值增长9%，社会消费品零售总额增长12%，地方财政收入增长8%，城乡居民人均可支配收入分别增长8.5%和9%左右，力争经济社会发展主要指标高于全省平均水平，走在关中前列。

重点做好八个方面的工作：

一、加快培育壮大产业集群，推动工业经济向中高端迈进

深入实施工业强市“1553”行动计划，做大做强存量、做新做优增量，努力提升工业质量和核心竞争力。

做大做强汽车产业。抢抓全省实施“300万辆汽车工程”机遇，围绕“一城两区五基地”目标，启动汽车产业发展行动计划。加快建设整车制造基地，支持吉利汽车争取再开工一条25万辆产能生产线，做好30万辆甲醇及新能源汽车前期工作；抓好陕汽宝华10万辆中轻卡、10万辆长头重卡以及10万辆通家新能源汽车项目建设，加强陕汽40万辆新能源轿车项目对接工作，扩大银河、宝石机械、通力等特种车产能。积极争取新的汽车名企落户，不断完善乘用车、商用车、专用车和新能源车产品体系，力争全年整车产销达到35万辆。加快建设关键零部件基地，支持吉利沃尔沃发动机一期36万台投产达效，抓好吉利宝鸡零部件产业园、法士特100万台中轻型变速器及新能源传动、陕汽汉德车桥30万根扩能技改、蔡家坡零配件园区等项目建设。积极引进新能源电池、电机、电控项目，争取力神电池项目开工。加快建设汽车及零部件检测基地，引进国内权威汽车整车检测机构和零部件检测中心，建设国家级整车和零部件检测基地。在高新区建立汽车产业计量中心，支持陕汽在蔡家坡建立产品中试中心。加快建设汽车生产性服务基地，联合国内外知名院校、科研机构、汽车企业等建立研发中心，筹建宝鸡汽车研究院及汽车学院。加快建设汽车文化产业基地，抓好蔡家坡汽车主题公园、陕汽汽车特色小镇等汽车文化综合体建设。与中汽协、中省体育部门等单位密切合作，常态化举办全国性汽车行业活动及赛事。年内整车及零部件产值达到1000亿元，2022年努力实现汽车产销100万辆，力争产能达到150万辆，总产值达到5000亿元，打响“丝路汽车名城”名片。

壮大特色优势产业。钛及新材料产业集群建设，要积极发挥钛产业联盟作用，着力突破钛材加工、增材制造等核心关键技术。抓好新材料公共检测服务平台、青海聚能钛业新材料循环再利用、高新区核级锆材等项目建设，支持宝钛老旧设备改造和生产线迁建。继续办好宝鸡·中国钛谷推介会和激光与钛产业合作论坛，推动钛产品迈向高端、步入生活。优势装备制造产业集群建设，要全面落实李克强总理“装备中国，走向世界”的指示精神，加快推动传统工业智能化改造，支持秦川机床机器人关节减速器、渭河工模具机器人谐波减速器等项目，抓好宝鸡机床智能化生产系统等建设，推动宝鸡制造向“宝鸡智造”迈进。积极融入高铁“走出去”战略，加快中铁电气化宝鸡科技产业园、中铁宝桥轨道交通产业园、中车时代扩能提升等项目建设。烟酒食品产业集群建设，要加快西凤酒城项目推进，完善包装、仓储物流、销售等产业配套，开发建设酒文化产业区。支持宝烟优化产品结构，不断做大做强。加快蒙牛纯甄生产线及高速灌装生产线项目建设，推动陇县、千阳高端羊乳全产业链发展。高端能化产业集群建设，要实施好郭家河煤矿二盘区开采工程，园子沟煤矿建成投用，加快90万吨甲醇项目进度，积极做好二甲醚及甲醇深加工、80万吨聚乙烯、2&times;1000兆瓦坑口发电等前期项目谋划，加速煤炭资源高端转化和循环利用。战略新兴产业发展，要加快7107航天基地、旋翼机产业园建设，推动无人机等高端精密项目产业化。抓好陇县华润关山风电、国电投凤县风力发电、麟游100兆瓦光伏农业二期等项目建设，组建宝鸡大数据中心，加快金台大数据、渭滨互联网产业孵化，推进紫光医药、扶风生物科技等产业园建设，尽快形成发展新动能。

加快推进军民融合。围绕建设国家级军民融合创新示范区，推进军地、军民全方位深度融合发展。建立军民融合联席会议制度，制定“军转民”项目目录和“民参军”产品技术目录，设立军民融合发展专项基金，鼓励军地组建联合研发团队，支持开展军民两用技术联合攻关。全面抓好总投资22.7亿元的53个军民融合重点项目建设，着力打造电子通讯、航空装备、新材料等三个军民融合产业园。主动加强与全国13个军工集团的精准对接，争取更多的项目布局宝鸡。

大力发展非公经济。落实各项支持政策，不断增强民营企业投资和发展信心。放宽市场准入，切实消除显性和隐性障碍，继续深化“联企”活动，加强金融信贷政策支持，年内培育规上企业50户，新增小微企业5000户。深入构建亲清新型政商关系，尊重企业和企业家，激发和保护企业家精神。继续推进高新区、蔡家坡等重点工业园区标准化厂房建设，进一步提升县域工业园区配套服务能力。围绕特色产业和骨干企业，延长产业链条，加快形成各具特色、优势明显的产业聚集区。

二、加快人文要素资源凝聚，促进文化旅游协同发展

传承历史文脉，挖掘文化内涵，以文强旅、以旅兴文，持续增强文化旅游影响力和支撑力。

做火宝鸡旅游。深入推进国家全域旅游示范市创建，大力实施旅游“5+”战略，抓好西周文化景区、大美佛汤城、天台山大景区、金台观三期、大东湖景区、扶眉战役纪念馆扩建等37个精品旅游项目，提升法门寺和太白山5A级景区建设水平，推进关山草原、大水川、青峰峡创建5A级景区。支持太白、凤县5A级景区县城建设，努力把太白县打造成中国达沃斯。积极发展航空、极限、冰雪、房车露营等前沿时尚的户外运动项目。筹建宝鸡市旅游发展投资公司，成立旅游产业发展基金。推进旅游交通体系、智慧旅游标识体系、公共服务设施体系建设，加强旅游形象策划、品牌塑造和宣传营销，不断提高宝鸡旅游知名度。力争全年接待游客9500万人次，实现旅游综合收入715亿元。

做活宝鸡文化。实施文物“活化”工程，抓好周原国家考古遗址公园建设，加快筹建工业遗址公园，按照世界文化遗产标准保护开发好周原和大雍城遗址，提升宝鸡特色文化的国际影响力。加快国家级周秦文化产业示范区、大秦帝国影视基地建设，力争市文化艺术中心建成开放。突出天地社火、凤翔泥塑、马勺脸谱等文化产品开发，加大非遗传承保护，更好地留住文化记忆。持续深化文化体制改革，加大资金投入，保障文化企业健康发展。积极探索“互联网+文化”，布局和打造宝鸡数字文化产业园，建立文化产品网络交易平台，提升文化产业发展水平。

讲好宝鸡故事。实施文化“走出去”战略，积极参加全省“国风·秦韵”“欢度春节”等国际巡演和“春雨工程”边疆行活动，打造《一号文件》电视剧等一批文化精品力作，举办系列文物外展活动，让“宝鸡文化”在国际国内舞台绽放魅力。弘扬宝鸡美食文化，在丝绸之路沿线国家和国内大中城市设立一批宝鸡美食餐馆。加快陕西省第九届艺术节筹备工作，抓好演出场馆建设和提升改造，启动宝鸡大剧院项目。深入开展各类群众性精神文明创建活动，持续增强市民认同感、归属感和向心力。

三、加快提升综合服务功能，增强城市辐射带动能力

实施城市品质提升“十大行动”，强化要素聚集、功能完善和服务保障，努力使宝鸡成为宜居宜业，让本地人自豪、外地人向往的美丽幸福城市。

全面提升城市品质。启动新一轮城市总体规划修编，高标准编制《宝鸡城市总体设计》，开展城市风貌研究，实施最严格的规划管控。加快植物园、阳平、联盟、团结、钛谷、清溪、陈仓、虢磻等8座渭河大桥，以及虢磻路跨陇海铁路立交桥、清姜路雨虹桥、金陵河中山桥、宝十桥头立交桥建设，力争斗中路高架桥快速干道及千河大桥开工，实施经福路等城市主干道路、背街小巷建设与提升改造，尽快构建“南北畅、东西快”的互联互通城市交通体系。高标准建设全国公交都市。力争城市轻轨车辆基地、车辆段、行政中心枢纽站等项目开工。抓好姜谭路综合管廊和宝福路等城市排水工程建设，加快老旧小区改造步伐，继续推进海绵城市试点和“双修”工作，努力提升城市现代化水平。

加速重点区域崛起。做强高新区，加快完善高铁新城基础设施，推进科技新城创新港及学校、医院建设，推动汽车及零部件、新材料、智能制造等优势产业聚集，努力打造产城融合、创新发展示范区。做大蔡家坡，抓好国家新型城镇化综合试点和省级小城市培育，加速形成市域副中心和汽车产业聚集区。建设新蟠龙，积极培育绿色产业和新兴产业，推动教育、卫生、文化、体育等优质资源向新区转移，加快建设幸福生态新城区。启动港务区，积极对接陕西自贸区，推进宝鸡综合保税区和阳平铁路物流中心建设，谋划建设宝鸡国际港务区。大力发展县域经济，强化产业支撑、园区承载、城镇带动，加快渭滨、金台、陈仓区向全省五强区迈进，凤县、凤翔、岐山在全省十强县中位次提升，扶风、眉县争当关中率先发展的“排头兵”，千阳、陇县、麟游、太白打造特色产业名县。持续推进汤峪、法门、柳林3个全国特色小镇和省级重点示范镇、文化旅游街区建设，打造一批工业重镇、商贸强镇、休闲名镇。

积极发展现代服务业。适应大众消费方式、消费热点和消费观念的转变，打造“制造+服务”模式，建设宝鸡金融文化商务区，发展会展经济，促进生产性服务业向专业化和价值链高端延伸。启动南客站吾悦广场和天下汇项目，继续推进东岭等城市综合体建设，打造代家湾地区商圈，提升胜利塬、太阳市等夜间经济示范点吸引力，促进生活性服务业向精细化和高品质转变。全力支持企业在宝鸡发展总部经济，培育共享经济、数字经济、现代供应链等新业态、新模式。

提升城市管理水平。进一步深化城管执法体制改革，整合各类基础资源，加强协同管理和服务，着力解决城市交通拥堵、交通安全等问题。深入开展市容秩序集中整治，提升保洁水平、绿化档次和夜景亮化品质，创建一批文明示范街区。继续加大城市“五拆”工作力度，整合腾退一批市区机关单位公共资产，全部用于建设绿地、停车场、健身休闲场所等市民服务设施。促进环卫精细化作业向背街小巷、城乡结合部等区域延伸。开展生活垃圾分类试点，加快垃圾发电项目建设。

四、加快实施乡村振兴战略，聚力打好脱贫攻坚战

按照产业兴旺、生态宜居、乡风文明、治理有效、生活富裕的总要求，促进农业生产方式、农民生活方式和农村生态环境不断改善，加快推进乡村振兴。

大力发展现代农业。突出质量兴农、绿色兴农，继续做好“三绿文章”、实施“三品工程”、推进“三产融合”，着力建设特色现代农业强市。组织开展产业结构调整大会战，切实保障粮食安全，加快千陇100万只奶山羊，扶风万亩猕猴桃，麟游、陇县万亩苹果，凤翔万亩中药材和全市6万亩苗木花卉基地建设，打造“世界猕猴桃之乡”“中国蜜都”、全国最大矮砧苹果基地和百亿羊乳产业。持续推进6个农产品加工园区建设，力争每个园区引进1-2个亿元以上加工企业。扩大陇县、千阳、太白绿色发展示范县创建试点成果，健全农产品质量追溯体系，认证无公害农产品、产地40个以上。加快建设现代农业产业园，打造斓翎溪谷、玉池公社、蜜蜂小镇、嘉陵绿谷等特色小镇和田园综合体。积极推进农业科技协同创新示范区建设，加强职业农民培育，促进农民专业合作社、农业产业化龙头企业和农业园区等新型农业经营主体多元融合发展。

全力推进精准脱贫。把提高脱贫质量放在首位，全面落实五年产业发展规划和年度增收计划，实施脱贫攻坚“十大行动”，不断壮大特色产业，因地制宜发展“小众”产业，持续深化电商扶贫、光伏扶贫、旅游扶贫等产业扶贫模式。注重发挥贫困群众的主体作用，把贫困户融入总体产业布局、嵌入产业链条，实现产业效益好、可持续。聚力23个深度贫困村，将民生项目、惠民政策最大限度地向贫困地区和贫困群众倾斜，基本完成易地扶贫搬迁和危房改造，进一步细化教育、健康、就业、生态和兜底扶贫政策措施，不断完善“普惠+特惠”政策保障机制，确保各项政策精准到户到人。做好扶志扶智工作，实现贫困户就业技能培训全覆盖。深化苏陕扶贫协作，完善中省单位“两联一包”、国企合力团等结对帮扶机制，提高扶贫实效。年内实现太白、麟游、千阳、扶风等4个贫困县摘帽、340个贫困村退出、7.1万贫困人口脱贫。

着力改善农村条件。实施水电路基础设施“三提升”行动，加快农村电网、气网改造升级与覆盖，完成农村公路“最后一公里”扫尾工作，力争“四好”农村公路建设走在全国前列。启动10个农田水利示范园区建设，加快普化、石沟、龙王河、南峡沟水库等重点水源工程建设，新修改造基本农田4万亩，治理水土流失面积400平方公里。加强土壤污染防治和农村面源污染治理，支持扶风、岐山开展秸秆综合利用试点，加快推进畜禽禁养区划定。继续抓好省级农村环境综合整治项目，建立长效管理机制，不断提高美丽乡村建设水平。

五、加快融入“一带一路”建设，全面构筑对外开放高地

着力构建现代化、立体式综合物流枢纽，更高水平引进来,更大力度走出去。

巩固交通枢纽地位。加快宝鸡至坪坎、太白至凤县、凤翔至旬邑高速公路建设，确保宝汉高速陇县至陕甘界、连霍高速宝鸡过境线、绛帐至汤峪高速路建成通车，力争眉县至太白、麟游至法门寺、关中环线宝鸡段高速路开工。推进西安—汤峪—法门寺—咸阳机场城际铁路建设，积极开展法门寺—宝鸡城际铁路前期工作，争取宝汉巴南铁路、宝平中卫铁路复线列入国家规划，力促宝鸡机场年内开工。抓好陕甘界至凤翔、两亭至良舍等14条国省干线公路升等改造。支持麟游、眉县、凤县、陇县规划建设通用航空机场，积极发展现代临空产业经济。

加快对外开放步伐。健全对外开放平台。推进电子口岸“单一窗口”推广应用，提高通关报检网络化、便利化水平。加强与国内外友好城市、丝路沿线城市、招商重点城市交流合作。不断扩大对外贸易。建好石油装备、钛材、猕猴桃等10个省级外贸出口示范基地，搭好“长安号”等6个过境货运专列“顺风车”，努力扩大外贸出口。深入推进“海外宝鸡”行动计划，加快红旗民爆、宝鸡机床等境外投资项目建设，在哈萨克斯坦、俄罗斯、印度等国家设立海外宝鸡展馆。建设俄罗斯冰淇淋宝鸡工厂及俄罗斯村，促进海外企业“走进来”。加大招商引资力度。紧盯全球产业链构建，以及国内重点地区产业转移，继续开展招商季活动，力争在大项目、工业项目引进上实现新突破。成立市招商服务中心，组建10个产业招商分局、4个驻外招商分局，健全区域中心城市宝鸡商会，举办宝鸡发展大会，推动招商工作常态化、专业化、精准化。各县区、开发区年内都要引进2至5个10亿元以上产业项目，全年招商引资到位资金增长15%以上。

壮大现代物流产业。构建物流园区、专业中心和分拨中心，以及铁路港、公路港和空港相结合的现代物流体系。加快吉利泰华物流产业园、西凤现代物流园建设，积极引进国内外知名物流企业在宝鸡设立总部或者分支机构，通过优化自营物流、物流外包和第三方物流，促进装备制造、农产品、汽车和大宗商品物流产业发展。加快创建国家电子商务示范市，大力发展物流配送等新型流通方式。通过现代物流理念和新技术改造提升传统物流业，力争年内创建一批国家现代物流示范园区。

六、加快推进美丽宝鸡建设，持续提升生态宜居水平

践行“绿水青山就是金山银山”理念，走绿色低碳发展之路，努力打造天蓝、地绿、水清的美丽宝鸡。

全域增绿。抢抓国家启动大规模国土绿化行动机遇，加快绿色宝鸡建设，培育健康稳定的森林生态系统。积极实施新一轮退耕还林、天然林保护和防护林工程，抓好渭河北坡绿化提升和保护修复，护山育林增加绿量。划定生态保护红线，加大秦岭生态保护力度，加强开山采石、河道采砂专项整治与供给保障。以“城市绿荫行动”、森林城市、森林特色小镇、城郊森林公园建设为重点，加快城乡绿化美化、提质增效，力争全年完成植树3500万株。

铁腕治霾。继续坚持源头严防、过程严管、质量严考、后果严惩的“四严”措施，启动新一轮三年保卫蓝天行动。加快推进工业减排、散煤整治和挥发性有机物治理，提高扬尘管控水平，加大“五改”和“三告别”工作力度，推动治污降霾向城乡共治展开。大力推进电、气清洁能源替代，统筹做好燃气气源、管网和应急储气设施建设，加快推进中石油西气东输管线和宝汉输气管线接气工程，确保空气质量优良天数不断增加，稳居关中城市前列。

柔性治水。实施水利建设“十大工程”，统筹推进城乡供水一体化、农田水利园区化、水域生态景观化。全面落实“河长制”“湖长制”，加快科技新城、蟠龙新区和五丈原污水处理厂建设，加强清姜河、金陵河、石坝河等重点支流污染治理。抓好太公湖等10个湖泊和100个生态涝池建设，建成扶风七星河、岐山落星湾、凤翔雍城湖等一批国家湿地公园。持续推进渭河市区段生态治理，实施渭河生态带水稻、油菜等特色观光农业项目，不断提升“百里画廊”建设水平。

七、加快改革创新步伐，进一步激发经济新动能

积极适应新时代高质量发展的需要，充分发挥改革创新对发展的支撑引领作用，不断激发新活力、释放新动能。

深化供给侧结构性改革。围绕优化供给结构和质量，完善要素市场化配置改革，重点在“破”“立”“降”上下功夫。围绕国家宏观政策和产业方向，破除无效供给，优化产业结构，杜绝和淘汰落后产能。持续改造提升建材、纺织服装、有色冶金三大传统特色产业，加快新旧动能转换，形成新的经济增长点。认真落实各项惠企政策，不断减轻企业税费负担，扩大直购电交易范围和规模，着力降低企业用能、物流、融资成本。

加大体制机制改革力度。深化国有企业改革，推广宝钛“1+N”改革模式，大力发展混合所有制经济，引进战略投资商参与宝鸡医药集团等一批国企整合重组。积极促进市城投公司实体化运营。规范市属国有企业薪酬管理，完成国企“三供一业”改革任务。统筹推进各类机构和扩权强县改革，赋予县区更多的发展自主权。建立透明、规范、高效的固定资产投资管理新机制。加大园区管理体制改革力度，探索多元化运营模式。强化农村集体产权制度改革，全面落实“第二轮土地承包到期后再延长三十年”政策，稳步推进土地所有权、承包权、经营权“三权分置”，探索“两权”抵押融资办法，农村“三变”改革实现全覆盖。抓住农村土地征收、集体经营性建设用地入市、宅基地制度改革试点范围扩大和耕地占补平衡管理办法改进的机遇，让更多的改革红利惠及“三农”。

着力推进创新驱动发展。坚持创新链、资本链、产业链和人才链融合发展，大力实施科技创新“十大工程”，积极培育一批科技型和高新技术企业，建设全省工业转型升级和提质增效先行示范区。加快国家区域创新中心、创新型科技园区和产业技术联盟布局建设，支持大企业集团在科技资源富集区建设协同创新软平台。继续开展“院士宝鸡行”“企业高校行”等产学研对接活动，柔性引进一批高端科技创新领军人才。抓好总投资338.6亿元的100个工业技改项目建设。支持秦川机床等国有企业开展“双创”，加大“众创空间”和孵化基地建设力度，扶持发展一批科技小巨人企业和瞪羚企业。

提高财政金融服务实体经济能力。加强财政资金整合，集中使用各类专项资金，探索推进供给方式改革。做大政府投资基金规模，做强汽车产业、科技创投、工业发展、基础设施建设等基金，吸引撬动更多的社会资本投入产业发展。组建消费金融公司和市水利发展投资公司，成立市小额贷款公司和融资租赁公司，设立中小微企业风险补偿基金，市中小企业融资担保公司注册资本金达到15亿元。支持西凤酒等企业在主板、新三板上市融资，建设100户上市后备企业资源库。全市社会融资规模存量达到2000亿元以上。切实抓好风险防控，严控政府性债务规模，加强小额贷款公司、融资担保公司和交易场所监管，深化互联网金融专项整治，严厉打击、化解变相非法集资等违法违规金融活动。加快社会信用体系建设，着力营造良好的金融生态环境。

八、加快完善公共服务体系，不断满足群众美好生活需要

坚持以人民为中心的发展思想，从群众最关心的事情做起，打造温情社会保障体系，使广大群众的获得感、幸福感、安全感更加充实。

以就业创业创造幸福。深入实施高校毕业生就业创业促进计划，支持返乡下乡人员创业创新，千方百计扩大就业。深化就业扶贫协作，探索建设一批社区工厂和就业扶贫基地，实现贫困劳动力充分就业。全年城镇新增就业4.5万人，转移农村劳动力85万人。

以社会保障巩固幸福。进一步落实全民参保计划，巩固扩大各类保险覆盖面。推进社会福利、未成年人保护等民生项目建设，切实加强住房保障，积极培育住房租赁市场。大力实施五大助残关爱工程，完善特困人员、孤寡老人、孤儿等社会救助制度，做好双拥优抚安置工作，加快养老服务市场化、产业化步伐。

以公共服务促进幸福。加大城区中小学、幼儿园建设力度，新建和改扩建68所学校，逐步消除义务教育学校大班额，提高普惠性幼儿园占比。加快宝鸡大学建设，高水平高质量普及高中阶段教育。建设市职业教育公共实训中心，打响“宝鸡技工”品牌。深入开展健康城市“十大行动”，启动市中医医院扩建，推进西安交大蟠龙医院项目，加强城市紧密型医联体、县域医共体、专科联盟等建设，健全分级诊疗体系，完善支付制度改革，提升医改“宝鸡模式”。优化计划生育服务,提高妇女儿童保健水平。加快全民健身场地设施建设。以承办全国第十四届运动会比赛项目为契机，继续办好国内高水平赛事。

以社会治理呵护幸福。提高社会治理法治化、智能化、专业化水平，构建共建共治共享的社会治理格局。实施从生产到消费全过程监督，打造国家食品安全示范城市升级版。切实加强事中事后监管，着力营造安全放心消费环境。加强和完善城乡社区治理，强化防灾减灾体系建设。积极稳妥做好第十次村委会换届工作。落实“七五”普法规划，构建惠民便民的公共法律服务体系。加强法治信访建设，严格信访工作责任制，落实社会稳定风险评估制度，解决好信访突出问题和群众合法诉求。以钉钉子精神强化社区警务，强力推进扫黑除恶专项斗争，打造全省最安全城市。进一步完善责任体系，创新安全监管方式，为人民群众提供更有保障、更可持续的安全感。

各位代表，人民对美好生活的向往就是政府工作的不懈追求，今年继续从群众诉求最为强烈的地方入手，着力办好十件实事。一是投资1.08亿元，开展第三期学前教育行动计划，实施高中阶段教育普及项目，改善城乡教育教学及师生生活条件。二是投资9200万元，完成230万城乡居民家庭医生有偿服务包的签约工作，不断提高群众健康水平。三是投资25亿元，新开工棚户区改造2万套，进一步解决好困难群众住房问题。四是投资5.92亿元，改善提升248个村68.92万人饮水条件，促进农村供水城市化。五是投资8亿元，加快“电化宝鸡”步伐，让更多居民享受供电优惠政策和优质服务。六是投资5亿元，在市区新建5000个停车位，持续缓解停车难问题。七是投资3000万元，继续开展技能培训全覆盖惠民工程，免费开展就业创业培训3万人。八是投资5600万元，推进33个社区服务中心、6个老年人日间照料中心和17个农村公益性公墓建设。九是大力实施“厕所革命”，逐步解决城市如厕难、农村难如厕问题。十是开展公益惠民演出1000场次，覆盖全市每个镇村，将文化大餐送到群众家门口。

各位代表，新时代新一年，加快宝鸡发展使命光荣、责任重大，对政府各项工作提出了新的更高要求。我们必须进一步解放思想，以舍我其谁的使命感、时不我待的紧迫感，加速追赶超越，创造无愧于时代、无愧于人民的新业绩。要突出项目带动，把抓项目作为转方式、调结构、增后劲的基础性工作和关键环节，着力解决新常态下宝鸡发展不是缺项目，而是缺谋划；不是缺资金，而是缺手段；不是缺资源，而是要学会如何利用资源的问题，将工作创新的要求转化为具体有形的项目，推动重点工作项目化、项目工作具体化，确保507个市级重点项目建设任务全面完成。要优化营商环境，深入推进以“最多跑一次”为重点的“放管服”改革，加快智慧城市建设，推进政务服务标准化，凡是网上能办理的决不跑窗口，凡是窗口能受理的决不跑部门，努力让群众、企业办事像“网购”一样方便。扎实开展营商环境提升年活动，推进优化提升营商环境十大行动，努力打造国际化、法治化、便利化营商环境。要强化责任担当，牢固树立“功成不必在我、事不竟我有责任”的观念，用好“三项机制”，增强各级干部真抓的实劲、敢抓的狠劲、常抓的韧劲，确保政府工作报告确定的目标任务年初建账、年中查账、年底交账，做到有言必行、有诺必践、有行必果。要依法廉洁从政，始终坚持依法行政，加快建设法治政府，自觉接受人大、政协和社会各界监督。不断强化“四个意识”，把纪律和规矩挺在前面，认真落实中央八项规定及实施细则，坚决反对“四风”，及时发现和严惩违纪违法行为，习惯在严明的纪律和严格的约束中工作生活，让清廉从政、清正干事成为政府工作的鲜明底色。

各位代表，时代的呼唤、人民的重托，激励着我们永不懈怠、奋力前行。让我们在市委的坚强领导下，以习近平新时代中国特色社会主义思想为指导，不忘初心、牢记使命，凝聚全市上下的智慧、才干和力量，一件事情接着一件事情办，一年接着一年干，为决胜全面小康做出新的更大贡献！
